# Case Report: Diagnostic pitfall and biomarker-guided therapy in CD20-negative Epstein–Barr virus-positive diffuse large B-cell lymphoma

**DOI:** 10.3389/fimmu.2026.1878880

**Published:** 2026-08-24

**Authors:** Jingjing Wen, Chenxing Zhao, Shaozhen Chen, Xiurong Chen, Qinghu Lv, Wei Liu, Jiesong Wang, Daoguang Chen

**Affiliations:** 1 Department of Lymphoma and Head & Neck Oncology, Clinical Oncology School of Fujian Medical University, Fujian Cancer Hospital, Fuzhou, China; 2 Department of Immunology, School of Basic Medical Sciences, Fujian Medical University, Fuzhou, Fujian, China; 3 Department of Nuclear Medicine, Clinical Oncology School of Fujian Medical University, Fujian Cancer Hospital, Fuzhou, China; 4 Department of Pathology, Clinical Oncology School of Fujian Medical University, Fujian Cancer Hospital, Fuzhou, China

**Keywords:** biomarker-guided therapy, CD20 negative, diagnosis, EBV positive DLBCL, individualized treatment

## Abstract

*De novo* CD20-negative Epstein–Barr virus-positive diffuse large B-cell lymphoma, not otherwise specified (EBV+ DLBCL, NOS), is a rare subtype of DLBCL. CD20 loss complicates both diagnosis and treatment by limiting anti-CD20 antibody use and broadening differential diagnosis. We report a case where comprehensive immunophenotypic and molecular analyses enabled accurate diagnosis. A biomarker-driven strategy combining CD30-targeted therapy and PD-1 blockade achieved a complete metabolic response. This case underscores the importance of biology-based individualized treatment in EBV-associated lymphomas and the need for prospective studies of CD20-independent strategies in high-risk patients.

## 1 Introduction

Epstein–Barr virus-positive diffuse large B-cell lymphoma, not otherwise specified (EBV+ DLBCL, NOS), is a distinct aggressive large B-cell lymphoma defined by Epstein–Barr virus (EBV)-encoded RNA (EBER) positivity in tumor cells and frequent immune-evasion features (1). As a subtype of DLBCL, EBV+ DLBCL is clinically and biologically heterogeneous. Its incidence varies geographically, accounting for approximately 3–5% of DLBCL cases in Europe and up to 10–15% in Asia and South America. Although usually expressing B-cell markers, some cases show aberrant immunophenotypes that obscure lineage assignment. *De novo* CD20-negative DLBCL is rare, accounting for less than 1% of all cases. It includes several specific entities, such as anaplastic lymphoma kinase-positive large B-cell lymphoma (ALK-positive LBCL) and plasmablastic lymphoma (2–4). In contrast, reduced or heterogeneous CD20 expression is more often seen in clinical practice. This pattern may be present at initial diagnosis or may emerge after anti-CD20 therapy as a mechanism of acquired resistance. CD20 loss or reduced expression can complicate both diagnosis and treatment, as it limits the use of anti-CD20 antibodies and broadens the differential diagnosis (5).

Here, we report a case of CD20-negative EBV+ DLBCL, NOS, with complete loss of conventional pan-B-cell markers (CD20, CD19, and CD79a) while retaining B-cell-specific transcription factors (PAX5, OCT2, and BOB.1). The patient was initially misdiagnosed with peripheral T-cell lymphoma, as the loss of pan-B-cell markers, coupled with anaplastic morphology and CD30 positivity, raised the possibility of a non-B-cell neoplasm. The diagnostic dilemma was further compounded by the absence of T-cell receptor gene rearrangement and the presence of diffuse EBER positivity, which required a comprehensive reassessment. Ultimately, repeat biopsy and integrative evaluation of immunohistochemistry, *in situ* hybridization, and immunoglobulin gene rearrangement analysis enabled correct lineage assignment and biomarker-guided treatment.

## 2 Case study

A 76-year-old male presented with cervical lymphadenopathy in August 2024. Prior to this presentation, the patient had been evaluated at an external institution for thrombocytosis in May 2022, during which bone marrow aspiration and myeloproliferative neoplasm (MPN)-related genetic testing were performed; JAK2 and TET2 mutations were identified, and a diagnosis of essential thrombocythemia (ET) was established. Intermittent hydroxyurea therapy was subsequently administered. And had other medical history included hypertension, diabetes, and aortic dissection treated with thoracic endovascular aortic repair. He had also undergone resection of basal cell carcinoma of the nasal cavity. He had no history of immunosuppression or transplantation. Physical examinations showed several enlarged lymph nodes in the cervical and left inguinal regions. Initial labs revealed: hemoglobin 13.3 g/dL, leukocytes 13.88×10^9^/L, platelets count of 1010×10^9^/L; LDH elevated at 406 U/L; EBV DNA in peripheral blood was 6.81E4 IU/mL; β2-microglobulin 9.77 mg/L; An excisional biopsy of a left cervical lymph node performed at a local hospital showed diffuse proliferation of large atypical lymphoid cells. Tumor cells were positive for CD30, BOB.1, and granzyme B, with partial expression of TIA-1, CD8, and CD25. Scattered cells expressed CD5. The neoplastic cells were negative for CD3, CD20, PAX5, OCT2, CD10, BCL2, BCL6, CD79a, ALK, CD15, TdT, CD4, CD7, CD56, and perforin. Ki-67 was approximately 90%, and EBER *in situ* hybridization was diffusely positive. On the basis of CD20 negativity, EBV positivity, and partial cytotoxic-marker expression, the case was initially diagnosed as peripheral T-cell lymphoma associated with EBV infection. A baseline 18F-FDG positron emission tomography-computed tomography (PET-CT) scan was then conducted for staging. PET/CT imaging revealed widespread, intensely hypermetabolic lymphadenopathy above and below the diaphragm. And associated with multifocal hypermetabolic lesions splenomegaly. Based on the diagnosis, the patient received one cycle of a dose-modified CHP (Cyclophosphamide, Doxorubicin, Prednisone) regimen (miniCHP) on August 2024. However, the lymphadenopathy did not show significant regression.

After referral to our center in September 2024, the original biopsy specimen was reviewed. Molecular testing showed no T-cell receptor (TCR) gene rearrangement. On this basis, the original diagnosis was reconsidered and revised to EBV-associated lymphoproliferative disorder. Repeat lymph node biopsy was recommended to clarify the diagnosis and guide further treatment, but the patient declined and chose to hold off on any additional therapy.

**Figure 1:**
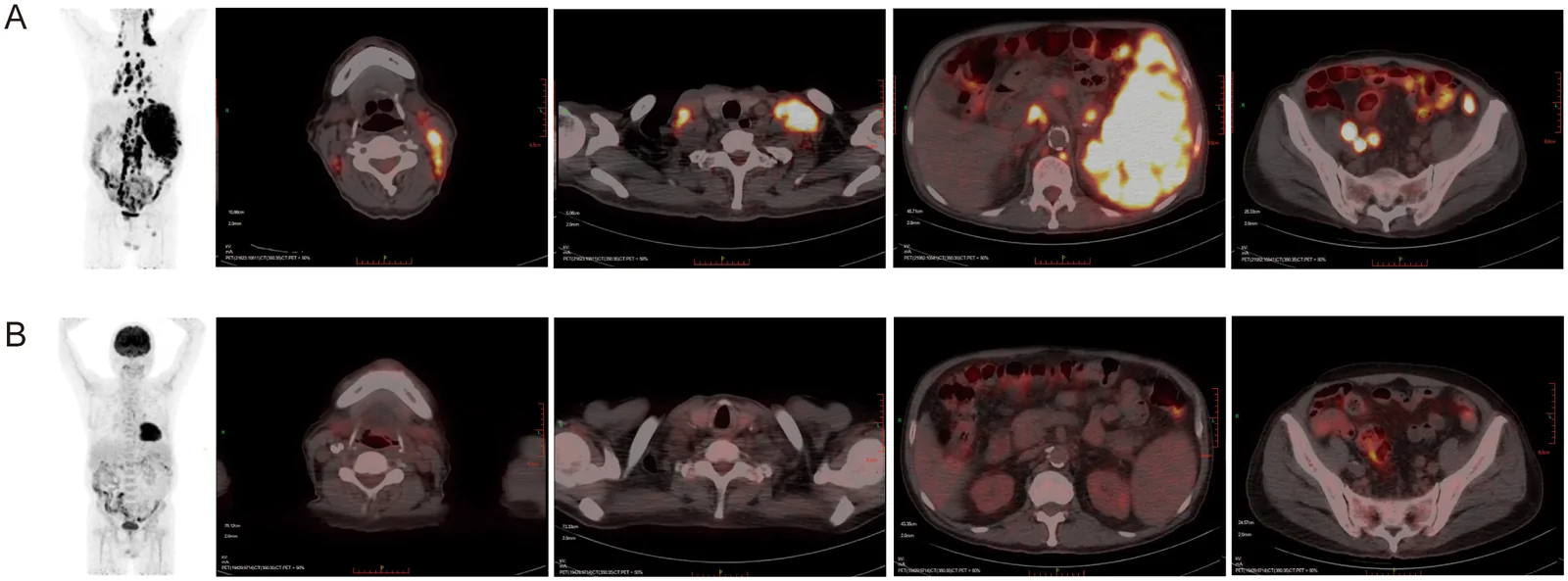
**(A)** The whole-body PET-CT February 6, 2025, revealed active disease progression. **(B)** A post-treatment whole-body PET-CT evaluation after 6 cycles treatment.

In November 2024, he developed progressive fatigue and malaise, accompanied by enlargement of multiple nodal masses, the largest measuring 4×3 cm in the left inguinal region. Follow-up PET/CT performed on February 6, 2025, demonstrated clear disease progression (**Figure 1A**). Peripheral blood EBV-DNA had increased to 9.58×10^4^ IU/mL. A repeat excisional biopsy of a left cervical lymph node was then performed.

**Figure 2:**
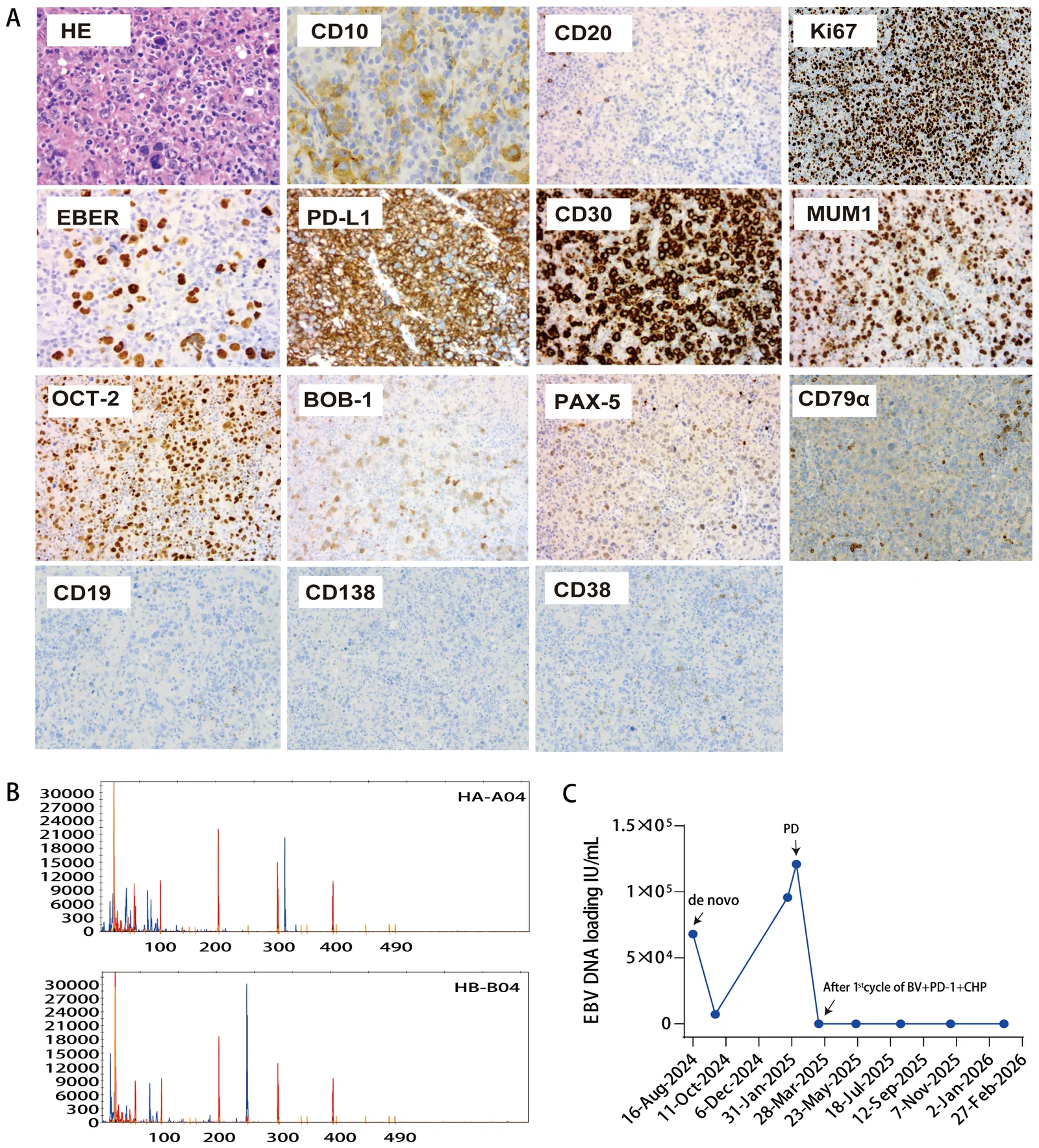
The histological characteristics of biopsy of left cervical lymph node. Light microscopy demonstrated diffuse growth of medium to large lymphoid cells on hematoxylin and eosin (HE) staining and immunohistochemistry of CD10, CD20, Ki67, PD-L1, CD30, MUM-1, CD79a, CD19, OCT-2, BOB-1, CD19, CD38, CD138 and *in situ* hybridization of EBER of second-time biopsy was shown on **(A)**. The BCR test of second biopsy was shown on **(B)**. The dot plot **(C)** of tendency of EBV DNA copy number at different treatment time points. PD, Progress disease, BV+PD-1+CHP: Brentuximab Vedotin combined with CHP (Cyclophosphamide, Doxorubicin, Prednisone) chemotherapy and Tislelizumab (an anti-PD-1 monoclonal antibody).

The second biopsy established the diagnosis of EBV+ DLBCL, NOS, CD20-negative variant. The diagnosis was supported by evidence of monoclonal B-cell proliferation with immunoglobulin gene rearrangement which showed a clonal rearrangement with distinct dominant peaks detected in the FR1-JH (HA-A04 reaction tube) and FR2-JH (HB-B04 reaction tube) (**Figure 2B**). What’s more, the second time biopsy showed an absence of T-cell receptor rearrangement, diffuse strong EBER positivity, loss of conventional pan-B-cell markers (CD20, CD19, and CD79a), retention of B-cell transcription factors (PAX5, OCT2, and BOB.1), and anaplastic morphology with uniform strong expression of CD30 and PD-L1 (**Figure 2A**). Based on the patient’s unique pathological profile. He received an individualized treatment strategy, consisting of Brentuximab Vedotin (BV) combined with CHP chemotherapy and Tislelizumab (an anti-PD-1 monoclonal antibody) for 6 cycles. And in total for 7 cycles including first cycle of miniCHP. The treatment was well tolerated, with no grade 4 immune-related adverse events or unexpected toxicities. End-of-treatment ^18^F-FDG PET/CT showed complete response (Deauville score 3) according to the Lugano 2014 criteria, with no residual hypermetabolic disease (**Figure 1B**). At the last follow-up in April 2026 (10 months after completion of therapy), the patient remained in remission. Serial monitoring of plasma EBV-DNA showed a progressive decline in parallel with clinical and metabolic response (**Figure 2C**).

## 3 Discussion

EBV+ DLBCL, NOS, represents a biologically heterogeneous and clinically aggressive subtype of DLBCL, predominantly affecting elderly patients (6). Despite being classified as a B-cell neoplasm, EBV+ DLBCL frequently exhibits atypical immunophenotypic features that may obscure lineage assignment and complicate initial diagnosis (7). The present case highlights several critical diagnostic and therapeutic challenges associated with this entity, particularly in the context of CD20-negative disease.

This unusual immunophenotype broadens the differential diagnosis and requires careful exclusion of other EBV-associated or CD20-negative large-cell lymphoma (1). Plasmablastic lymphoma is an important consideration. However, the retention of B-cell transcription factors, including PAX5, OCT2, and BOB.1, together with the absence of diffuse CD138 expression, argues against a fully plasmablastic phenotype (8). Although classical Hodgkin lymphoma (cHL) shares anaplastic morphology and CD30/PD-L1 expression with this case, it was excluded because the tumor cells formed diffuse sheets rather than the sparse, inflammatory-rich pattern typical of cHL (9). Despite CD20 loss, the absence of CD15 and retention of PAX5, OCT2, and BOB.1 remains inconsistent with the profound B-cell lineage deficit seen in cHL (10). ALK-negative anaplastic large cell lymphoma may also enter the differential diagnosis because of the strong CD30 expression and large-cell morphology, but the presence of B-cell transcription factors and negative in TCR rearrangement doesn’t support this diagnosis (9). Chronic active EBV-associated T/NK-cell lymphoproliferative disorders also warrant exclusion, particularly when EBV-positive lymphoid cells are present. In this case, however, the overall morphology and immunophenotype favored an EBV-positive large B-cell process over a T/NK-cell lymphoproliferative disorder (11). Therefore, integration of morphology, EBV status, and an extended immunohistochemical panel was essential for establishing the final diagnosis of EBV+ DLBCL, NOS.

The diagnostic challenge is further compounded by the frequent loss of not only CD20 but also other pan-B-cell markers (CD19, CD79a, and occasionally PAX5) in CD20-negative EBV+ DLBCL, a phenomenon that may mimic various T-cell or null-cell lymphomas (2–4, 12). Recognition of this rare phenotype is clinically relevant, as loss of CD20 may limit the benefit of rituximab-containing therapy and may partly explain the aggressive behavior and treatment difficulty observed in some cases (12). The present case shares several of these features, including older age, EBV positivity, loss of conventional pan-B-cell markers, retention of B-cell transcription factors, and strong CD30/PD-L1 expression, highlighting both the diagnostic pitfalls and potential therapeutic implications of CD20-negative EBV+ DLBCL.

CD20 is a non-glycosylated phosphoprotein expressed on mature B cells and plays an important role in B-cell receptor signaling, calcium flux, and cell-cycle regulation. The success of rituximab established CD20 as a central therapeutic target in DLBCL (13). However, *de novo* CD20-negative DLBCL represents a rare and clinically challenging subtype for which rituximab-based therapy is inherently limited. The mechanisms underlying primary CD20 loss differ from secondary CD20 downregulation after anti-CD20 exposure (14). In DLBCL, absent CD20 expressions are often associated with plasmablastic or terminal B-cell differentiation, accompanied by loss of other pan-B-cell markers. Proposed mechanisms include epigenetic silencing of the MS4A1 promoter and dysregulation of transcriptional networks governing B-cell identity (15, 16).

Currently, R-CHOP remains the conventional first-line treatment for EBV+ DLBCL, although reported complete response rates vary widely, from approximately 30% to 70%. More recently, polatuzumab vedotin has expanded the therapeutic landscape of DLBCL. In the POLARIX study, pola-R-CHP improved progression-free survival compared with R-CHOP in the overall DLBCL population (2-year progression-free survival 76.7% vs. 70.2%) (17). However, its activity in EBV+ DLBCL has not been specifically established. In the present case, the lack of both CD20 and CD79a expression suggested that neither rituximab- nor CD79b-directed treatment would be optimal, reinforcing the need for a biomarker-driven strategy.

Given the absence of CD20 and CD79a, alternative therapeutic targets were therefore considered. CD30 expression is frequently observed in EBV-associated lymphomas and reflects constitutive activation of NF-κB and JAK/STAT signaling pathways (18). Brentuximab vedotin (BV), an antibody–drug conjugate targeting CD30, has demonstrated significant activity in CD30-positive lymphomas beyond classical Hodgkin lymphoma, including subsets of DLBCL. Although prospective data specifically addressing BV-based therapy in EBV+DLBCL are limited (19), emerging evidence supports its biological and clinical relevance, particularly in CD20-negative cases. In parallel, EBV-associated lymphomas are characterized by an immunosuppressive tumor microenvironment, frequently marked by high PD-L1 expression driven by EBV latent gene products and genetic alterations involving the 9p24.1 locus (20). The exceptionally high PD-L1 combined positive score observed in this case provided a strong biological rationale for incorporating PD-1 blockade. Recent studies have reported meaningful responses to PD-1 inhibitors in relapsed or refractory EBV-positive lymphomas (21), supporting their role as an effective therapeutic component in selected patients. The combination of BV with PD-1 inhibitor and CHP chemotherapy in this patient was therefore rationally designed, aligning with the biomarker-rich profile of this case and existing data on CD30-positive aggressive lymphomas.

In addition to the diagnostic complexities discussed above, this case presented another layer of clinical interest, the concurrent diagnosis of ET. The co-occurrence of ET and DLBCL represents a rare but increasingly recognized clinical entity, with epidemiological data demonstrating a significantly elevated risk of secondary NHL among MPN patients (22–24). The pathogenesis of this concurrent presentation is likely attributable to shared clonal origins arising from a common ancestral hematopoietic stem cell harboring somatic mutations in epigenetic regulatory genes, most notably *TET2* and *DNMT3A (*25, 26). And such mutations may drive myeloid and lymphoid oncogenesis. Furthermore, the JAK2 V617F mutation, present in approximately 55% of ET cases, confers constitutive activation of the JAK-STAT signaling pathway, which may exert oncogenic influence not only on myeloid progenitor cells but also on B-lymphocyte survival and differentiation, potentially facilitating lymphomagenesis (27, 28).Though the present case does not establish a causal relationship between the two conditions. Delineating the putative molecular mechanisms for this co-occurrence may provide novel therapeutic insights for the future clinical management of patients presenting with this rare combination of hematologic malignancies.

## 4 Conclusion

In summary, this case underscores that CD20-negative EBV+ DLBCL represents a formidable diagnostic pitfall, which can be navigated through systematic integration of morphology, extended immunophenotyping, and molecular clonality studies. Therapeutically, the durable complete response achieved with a biomarker-driven regimen, targeting CD30 and PD-L1 in the absence of CD20. Suggesting that alternative targeted strategies may offer meaningful benefit in this rare subset. Furthermore, the concurrent diagnosis of ET highlights the potential role of shared clonal origins in MPN-lymphoma co-occurrence. And the parallel decline of plasma EBV-DNA with treatment response supports its utility as a noninvasive monitoring biomarker. Although conclusions from a single case are inherently limited, this experience provides a pragmatic framework for the diagnostic and therapeutic management of similar challenging cases.

## Data Availability

The original contributions presented in the study are included in the article/supplementary material. Further inquiries can be directed to the corresponding authors.
